# Oocytes with a Dark Zona Pellucida Demonstrate Lower Fertilization, Implantation and Clinical Pregnancy Rates in IVF/ICSI Cycles

**DOI:** 10.1371/journal.pone.0089409

**Published:** 2014-02-24

**Authors:** Wei Shi, Bo Xu, Li-Min Wu, Ren-Tao Jin, Hong-Bing Luan, Li-Hua Luo, Qing Zhu, Lars Johansson, Yu-Sheng Liu, Xian-Hong Tong

**Affiliations:** 1 Center for Reproductive Medicine, Anhui Provincial Hospital Affiliated to Anhui Medical University, Hefei, China; 2 Centre for Assisted Reproduction, Codra Hospital, Podgorica, Montenegro; Clermont Université, France

## Abstract

The morphological assessment of oocytes is important for embryologists to identify and select MII oocytes in IVF/ICSI cycles. Dysmorphism of oocytes decreases viability and the developmental potential of oocytes as well as the clinical pregnancy rate. Several reports have suggested that oocytes with a dark zona pellucida (DZP) correlate with the outcome of IVF treatment. However, the effect of DZP on oocyte quality, fertilization, implantation, and pregnancy outcome were not investigated in detail. In this study, a retrospective analysis was performed in 268 infertile patients with fallopian tube obstruction and/or male factor infertility. In 204 of these patients, all oocytes were surrounded by a normal zona pellucida (NZP, control group), whereas 46 patients were found to have part of their retrieved oocytes enclosed by NZP and the other by DZP (Group A). In addition, all oocytes enclosed by DZP were retrieved from 18 patients (Group B). No differences were detected between the control and group A. Compared to the control group, the rates of fertilization, good quality embryos, implantation and clinical pregnancy were significantly decreased in group B. Furthermore, mitochondria in oocytes with a DZP in both of the two study groups (A and B) were severely damaged with several ultrastructural alterations, which were associated with an increased density of the zona pellucida and vacuolization. Briefly, oocytes with a DZP affected the clinical outcome in IVF/ICSI cycles and appeared to contain more ultrastructural alterations. Thus, DZP could be used as a potential selective marker for embryologists during daily laboratory work.

## Introduction

A fundamental and important part of daily in-vitro fertilization (IVF) laboratory work is the morphological assessment of oocytes and embryos, which guides the identification and selection of MII oocytes with the potential to develop into good quality embryos with high implantation potential [1], [2]. Dysmorphism of oocytes and embryos, such as irregular shape [3], dark cytoplasm [4], refractile bodies [5], a large second polar body [6], [7] and large perivitelline space [1], [8]–[10], affects the viability and developmental competence of pre-implantation embryos and consequently increases early pregnancy loss [11]–[13].

In addition, the morphological appearance and structural orientation of the zona pellucida (ZP) are predictive of the quality of the oocytes and embryos. Oocytes with defective ZP are correlated with decrease rates of blastocysts, implantation, and clinical pregnancy, and increases in the miscarriage rate [2], [14]. Moreover, the density, structure or interaction of the ZP glycoproteins are correlated with the quality of the follicles and could be used to predict implantation and miscarriage rates [15]. Taken together, these findings are correlated with the expression of specific genes in the cumulus-oophorous- complex (COC) [2], which in infertile mice with defective ZP, are dependent upon the lack of genes for ZP2 and ZP3 [16], [17]. In addition, variation in the thickness of the zona pellucida has been found to correlate with fertilization [18], [19], embryo quality and implantation rates [20], [21].

In contrast, the dark zona pellucida (DZP), as suggested by several reports, does not affect the fertilization, embryo quality or pregnancy rate [1], [22], [23]. In these studies, a mixture of embryos surrounded by both a DZP and normal zona pellucida (NZP) was transferred, which makes it impossible to determine which type of embryos (with a DZP or NZP) were implanted. In this study, the patients were divided into two different groups: 1) in the first group, not all of the oocytes were surrounded with a DZP (Group A); 2) in the second group, all of the oocytes were surrounded with a DZP (Group B). Thus, in the second group, only embryos surrounded with a DZP were transferred, enabling the determination of the effect of DZP on fertilization, implantation, embryo development and pregnancy outcome.

## Materials and Methods

### Ethics Statement

Patients were recruited among infertile couples seeking IVF/ICSI treatment at the Center for Reproductive Medicine, Provincial Hospital, Anhui, between October 2009 to March 2012. A written informed consent was obtained from all participants involved in the study. This study was performed according to the Declaration of Helsinki and the Ethics Committees on Human Research of Anhui Provincial Hospital, an affiliation of the Anhui Medical University (Approve ID: 2008010602).

### Patient Population

This research was a retrospective cohort study. A total of 268 infertile couples (age <38 years; tubal disorders and/or male factor) treated with either IVF or ICSI were recruited for the study. Of these patients, 64 patients were placed into the study group [41 IVF, 28 ICSI (3 patients with seven cycles) and 58 embryo transfer cycles; oocytes surrounded by DZP] and 204 patients [143 IVF, 63 ICSI (2 patient with four cycles) and 180 transfer cycles] in the control group (oocytes surrounded by NZP). The patients in the study group were subdivided into two groups according to the percentage of oocytes surrounded by DZP. Group A consisted of patients, where part of their oocytes were surrounded by DZP (33 IVF, 14 ICSI cycles and 38 transfer cycles), and in group A, the percentage of oocytes with a DZP was 58% on average (max: 78% and min: 17%) in one oocyte pick-up cycle. Group B consisted of patients, in which all of their oocytes were surrounded by DZP (8 IVF, 14 ICSI cycles and 20 transfer cycles). The exclusion criteria were uterine malformations, chromosomal abnormalities and a poor ovarian response.

### Stimulation Regimes

For all patients, a long pituitary down-regulation protocol was used. Briefly, a long-acting gonadotropin-releasing hormone agonist (GnRH-a, Diphereline; Ipsen Pharma Biotech, Signes, France) was injected intramuscularly in the mid-luteal phase of the preceding cycle of gonadotropin (Gn: rFSH, Gonal-F, Merk Serono SA, Geneva, Switzerland) stimulation. Afterwards, complete pituitary down-regulation injections of r-FSH were initiated. Human chorionic gonadotropin (HCG, LiZhu Pharma., ZhuHai, China, 10000 IU) was injected when at least two follicles had reached 18 mm in diameter or if more than three follicles had reached 17 mm in diameter. Thirty-six hours later, the hCG injection oocytes were retrieved via transvaginal aspiration of the oocytes. The decision on whether IVF or ICSI was to be performed was determined upon the quality of the semen on the day of oocyte retrieval.

### Sperm Preparation, Insemination Techniques and Control of Fertilization

The oocytes were cultured together in a large drop of fertilization medium (William A. COOK Australia Pty. Ltd, Queensland, Australia) under mineral oil in a 35-mm Petri dish (Falcon 1008; Becton & Dickinson, Lincoln Park, NJ). The semen samples were prepared via density gradient centrifugation [24]. If the number of motile sperm (a+b) in the semen sample was >10 × 10^6^, then the prepared sperm suspension was used for IVF or <10 × 10^6^ for ICSI. The oocyte was considered fertilized if a second polar body was extruded or if two pro-nuclei were observed 16 hours after insemination. Normally fertilized oocytes were transferred into fresh drops of cleavage medium (William A. COOK Australia Pty. Ltd., Queensland, Australia).

### Evaluation and Selection for Embryo Transfer (ET)

Embryo quality was evaluated between 42–48 hours after insemination classified according to the following principle: grade1 (even and homogeneous blastomeres without fragmentation cytoplasmic fragmentation), grade 2 (even and homogeneous blastomeres with <20% cytoplasmic fragmentation), grade 3 (uneven and non-homogeneous blastomeres with 20–50% cytoplasmic fragmentation), and grade 4 (uneven and non-homogeneous blastomeres with >50% cytoplasmic fragmentation). Embryos grade 1 and 2 were considered good quality embryos from which 2–3 embryos were selected for trans-abdominal ET under ultrasound guidance on Day 3. Surplus good quality embryos were vitrified. From the day of oocyte retrieval, the patient was administered daily intramuscular injections of progesterone (40 mg) on the initial three days and then progesterone (60 mg) on the following days (for approximately three months). Pregnancy was diagnosed using a positive blood test for hCG at 14 days after embryo transfer. A clinical pregnancy rate was considered established if a gestational sac with a fetal heart beat was found via a transvaginal ultrasound four weeks after the positive pregnancy test. All pregnancies were monitored until the time of delivery.

### Removal of Cumulus Cells and Observation of the ZP

In ICSI cycles, cumulus cells of the corona radiata were enzymatically removed with 40 IU/ml of hyaluronidase (SAGE In Vitro Fertilization, Inc. Trumbull, USA) in Hepes-buffered Gamete Medium (William A. COOK Australia Pty. Ltd., Queensland, Australia) using hand-pulled glass denudation pipettes. After denudation, the oocytes were transferred into a dish containing fresh Hepes-buffered medium. In IVF cycles, cumulus cells were removed using hand-pulled glass denudation pipettes after fertilization for 16–18 hours. Next, their zona pellucidas (from the ICSI and IVF cycles) were evaluated using an inverted microscope at 400× magnification.

### Evaluation of Oocytes using Transmission Electron Microscopy (TEM)

A total of twenty mature oocytes were included in this study. Ten oocytes with a normal zona pellucida (NZP; Control group) and ten oocytes with a dark zona pellucida (DZP, from Group A and Group B) were fixed for four hours after egg collection. The oocytes were fixed and processed for TEM analysis as previously described by Nottola et al., 2009 [25]. Ultrathin sections (60–80 nm) were cut using a diamond knife, and the sections were mounted onto a copper grid and contrasted with saturated uranyl acetate, followed by lead citrate prior to analysis and imaging (JEOL-1230 Transmission Electron Microscope).

### Statistical Analysis

Statistical analysis was performed using SPSS version 13 statistical software. Continuous data were described as the mean ± sd and compared using a one-way ANOVA test. The rates were compared using Chi square and Fisher’s exact test when appropriate. Differences in P-value <0.05 were considered to be statistically significant.

## Results

### Basic Clinical Information

No significant differences were found between the three groups in basic patient information (age, duration of infertility), sexual hormone levels, days of ovarian stimulation, doses of used gonadotropins, values of E2 and endometrial thickness on the day of HCG and the number of embryos per ET (Table 1).

**Table 1:** Basic Patient information.

Parameter	Control Group	Partial Oocytes with a DZP (Group A)	All Oocytes with a DZP(Group B)	P-value
**No. of cycles (IVF+ICSI)**	206(143+63)	47(33+14)	22(8+14)	–
**Age (year, mean±sd)**	30.25±4.05	29.77±3.87	31.45±4.16	0.268
**Infertility factors**	Tubal disorders and/or male factor	Tubal disorders and/or malefactor	Tubal disorders and/or malefactor	
**Duration of infertility (year, mean±sd)**	4.59±2.84	4.59±2.83	5.11±2.75	0.708
**bFSH (IU/L, mean±sd)**	7.21±2.37	7.89±2.66	7.56±2.53	0.21
**bLH (IU/L, mean±sd)**	4.56±2.12	4.28±1.91	3.88±1.80	0.294
**bE2 (pg/ml, mean±sd)**	50.16±24.23	56.27±27.65	50.87±27.41	0.352
**bPRL (ng/ml, mean±sd)**	14.59±6.64	15.26±5.47	12.18±4.77	0.187
**bT (nmol/ml, mean±sd)**	0.48±0.64	0.51±0.40	0.42±0.21	0.886
**Days of ovarian stimulation (mean±sd)**	12.97±2.41	13.36±2.83	12.95±1.89	0.584
**Total Gn doses(IU/L, mean±sd)**	2500.80±827.49	2757.45±951.99	2676.14±716.44	0.137
**E2 on HCG day (pg/ml, mean±sd)**	2816.58±1384.67	3040.37±1338.12	2816.58±1384.67	0.482
**Endometrial thickness on HCG day** **(mm, mean±sd)**	11.21±2.38	11.05±1.97	11.19±2.40	0.912
**No. of transferred embryos/cycle**	2.20±0.42	2.19±0.40	2.16±0.50	0.914

P<0.05 was considered statistically significant.

### Treatment Outcomes

**Figure 1:**
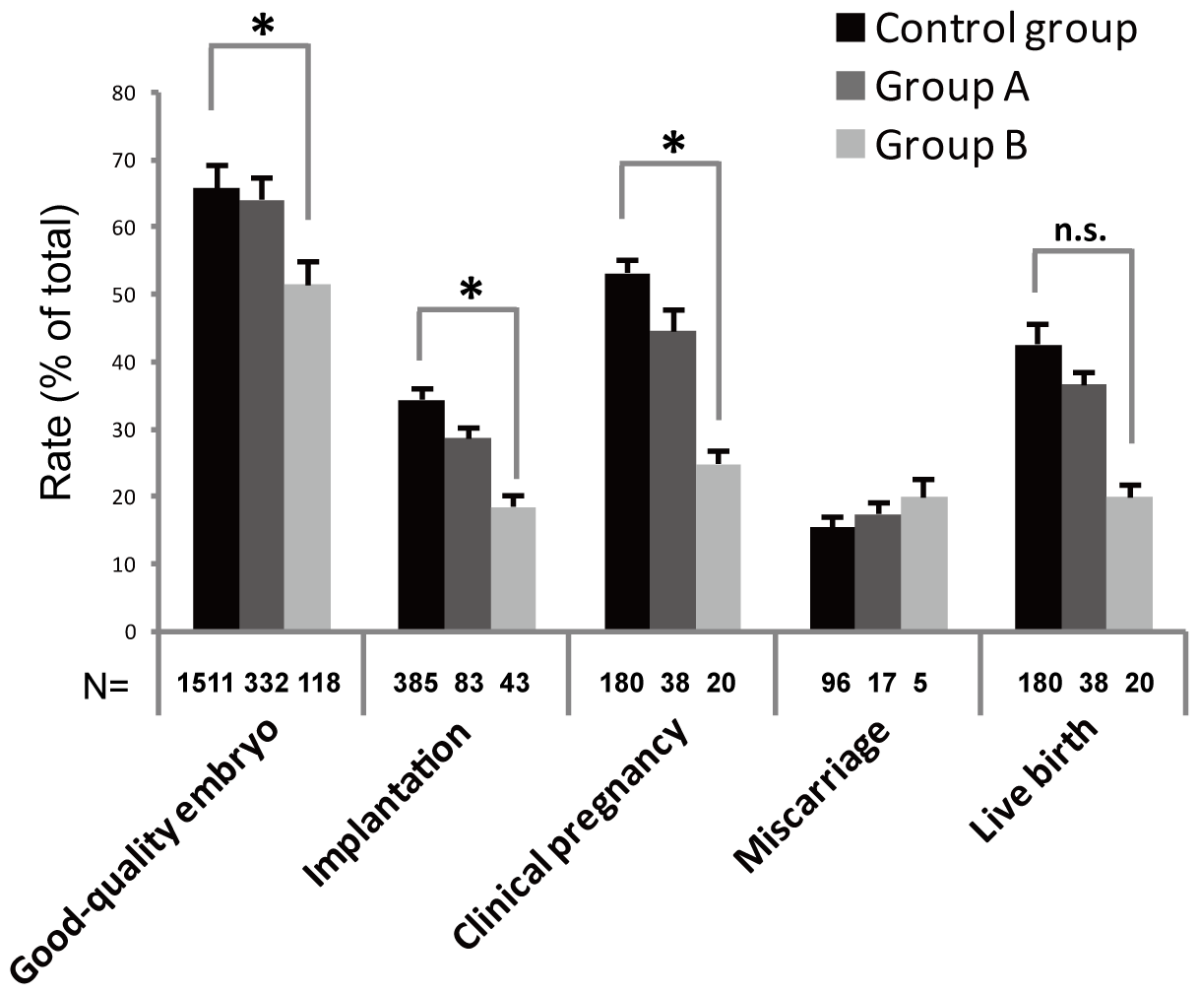
Pregnancy outcome of patients with a normal (Control), low (Group A) and high (Group B) frequency of oocytes with a dark zona pellucida. There were no significant differences between the three groups in terms of miscarriages and live births. However, the fertilization rates (IVF; 59.4 vs. 76.3%, resp. ICSI 67.7 vs. 82.3%), rate of top quality embryos (51.7 vs. 66.0%), implantation rates (18.6 vs. 34.5%) and clinical pregnancy rates (25.0 vs. 53.3%) was significantly different (*: P<0.0167) between the control versus Group B. The likelihood of the fertilization rate, rate of top quality embryos, implantation rate, and live birth rate after IVF/ICSI treatment and specific study factors (Control group, Group A, and Group B) was presented as the odds ratio with 95% confidence interval (CI). Each bar represents a 95% CI.

No differences were detected between the Control and Group A in the percentage of MII oocytes (80.5% vs. 81.0%, in ICSI cycles), normal fertilization rate (2PN) in IVF or ICSI cycles (68.6% vs. 76.3% and 81.6% vs. 82.3%, respectively) and cleavage rate (98.2% vs. 98.7%) (Table 2). A similar pattern was also detected in the proportion of good quality embryos (64.2% vs. 66.0%), implantation (28.9% vs. 34.5%), clinical pregnancy (44.7% vs. 53.3%), miscarriage (17.6% vs. 15.6%) and live birth rates (36.8% vs. 42.7%) (Figure 1).

**Table 2:** Number of retrieved and mature oocytes, and fertilization and cleavage rates.

Parameter		Control Group	Partial Oocytes with a DZP(Group A)	All Oocytes with a DZP(Group B)	P-value
**Rate of MII oocytes** **(%, ICSI cycle)**		81.0%(588/726)	80.5%(120/149)	73.7%(90/122)	0.179
**Normal fertilization** **(2PN) rate (%)**	**IVF**	76.3%(1046/1371)	68.6%(240/350)	59.4%(60/101)*	**P<0.001**
	**ICSI**	82.3%(484/588)	81.6%(98/120)	67.7%(61/90)*	**P<0.05**
**Abnormal fertilization rate (%)**	**IVF**	2.8%(39/1371)	4.0%(14/350)	4.0%(4/101)	0.242
	**ICSI**	1.0%(4/588)	0	2.2%(2/90)	0.169
**Cleavage rate (%)**		98.7%(1511/1530)	98.2%(332/338)	97.5%(118/121)	0.442

P<0.05 was considered statistically significant. Control group vs. DZP (Group B); *(P<0.0167).

NOTE: Rate of MII oocytes: MII oocytes/total oocytes in ICSI cycle; Normal fertilization rate: two-pronuclear zygotes (2PNs)/total MII oocytes in ICSI cycle or 2PNs/total oocytes in IVF cycle; Abnormal fertilization rate: 1PNs+multi-PNs/total MII oocytes in ICSI cycle or 1PNs+multi-PNs/total oocytes in IVF cycle; Cleavage rate: embryos on the 3rd day of cleavage–stage/total 2PNs; Rate of good quality embryos: good quality embryos/total embryos on the 3rd day; Implantation rate: total number of gestational sac/total embryos transferred; Clinical pregnancy rate: total number of clinical pregnancy/total transfer cycles; Miscarriage rate: total number of miscarriage/total number of pregnancy. Live birth rate: total man–time of live baby/total number of pregnancy.

The rates were significantly lower in Group B compared to those in the control group of normal fertilization (2PN) in IVF and ICSI cycles (59.4% vs. 76.3 and 67.7% vs. 82.3%) (Table 2), good quality embryos (51.7% vs. 66.0%), implantation (18.6 vs. 34.5%) and clinical pregnancy (25.0% vs. 53.3%) (Figure 1). However, there were no significant differences between the two groups in the percentage of MII oocytes (73.7% vs. 81.0%, in ICSI cycles), cleavage (97.5% vs. 98.7%) (Table 2), miscarriage (17.6% vs. 15.6%) and live birth rates (20.0% vs. 42.7%) (Figure 1).

### Micro- and Ultra-structure of Oocytes

**Figure 2:**
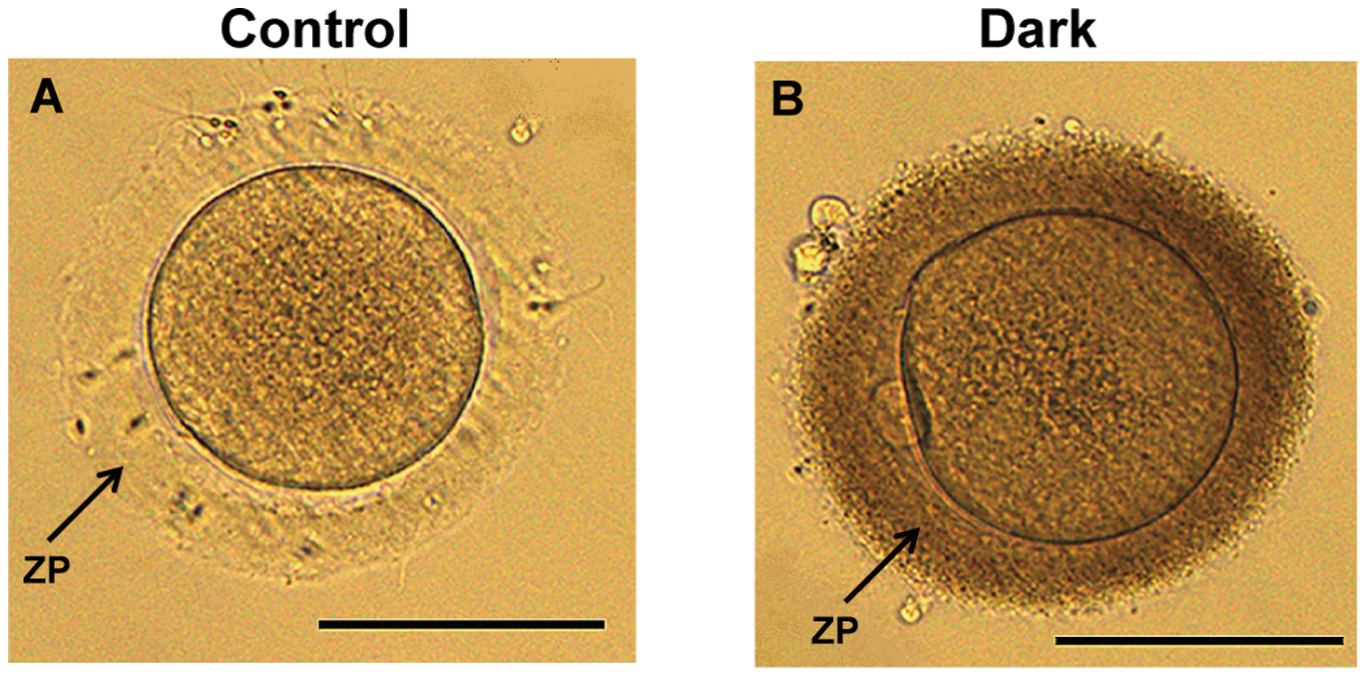
Human mature oocytes with a normal (A) and dark (B) zona pellucida. Scale bar (A, B): 100 µm.

**Figure 3:**
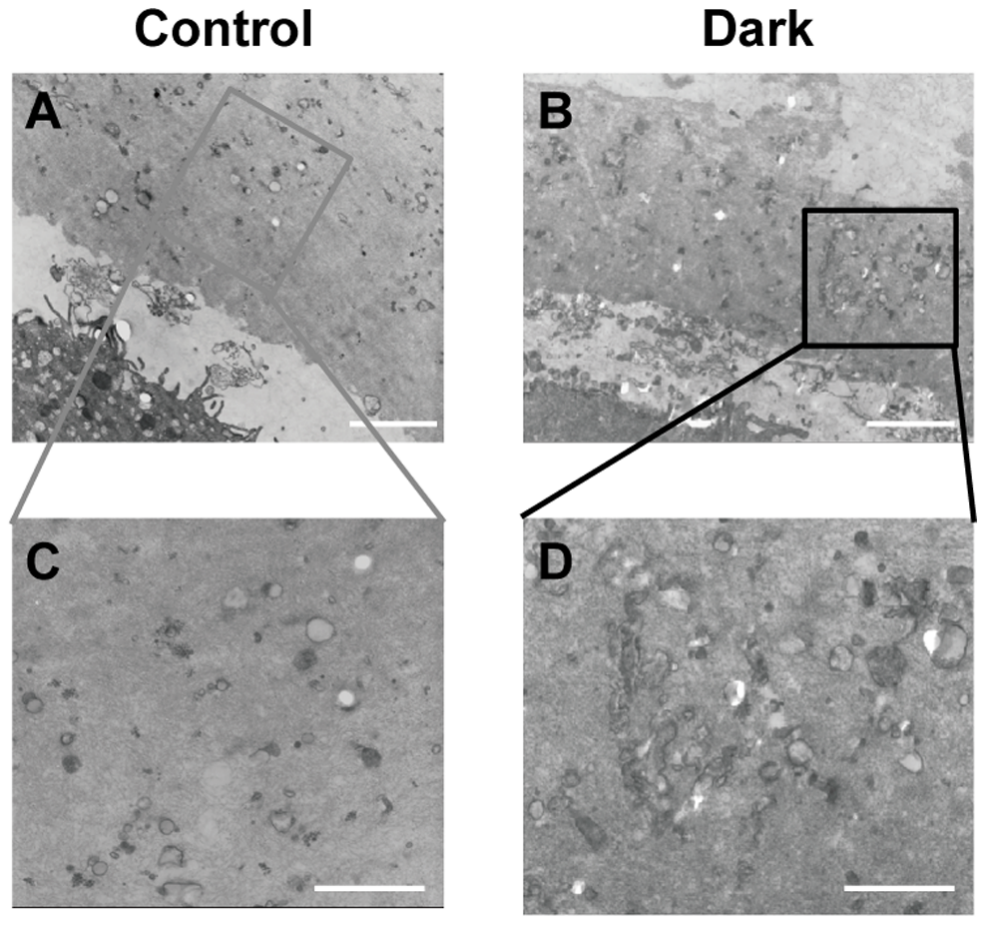
The electron density of normal (A, C) and dark zona pellucida (B, D). Scale bar (A, B): 1 µm. Scale bar (C, D): 500 nm.

Apparent differences were observed in the color of the zona pellucida (ZP) of oocytes surrounded by a normal (Figure 2A) or dark ZP (Figure 2B). First, we detected the thickness of the normal and dark ZP by measurement of the optical microscopy images; however, the thickness of the DZP was not increased compared with the NZP (Supplementary Fig. S1). Ultrastructural analysis using TEM revealed more closely packed electron-dense fibrillar materials present in the DZP (Figures 3B and D) compared to the NZP (Figures 3A and C).

**Figure 4:**
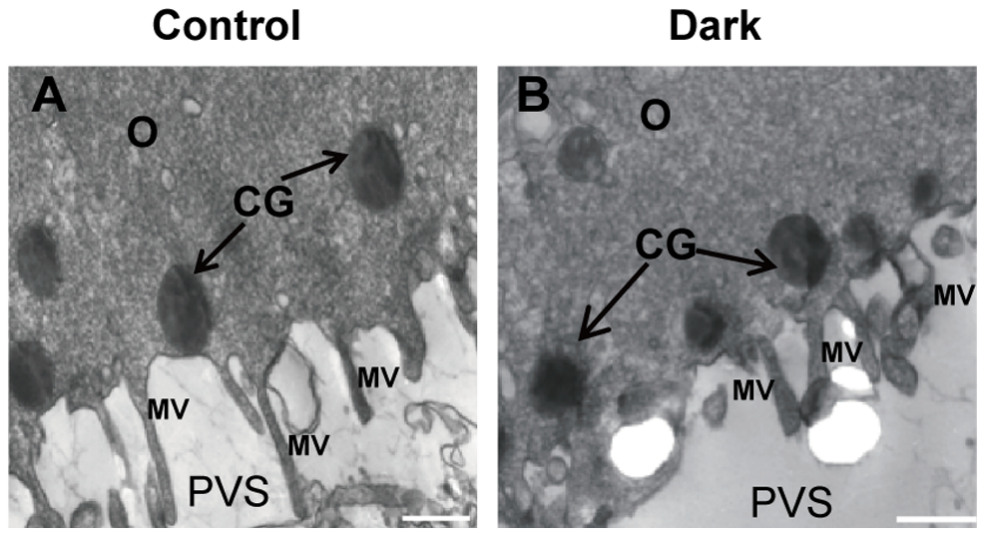
Dense cortical granules (CG) and microvilli (MV) in oocytes from control and oocytes with a dark zona pellucid. A rim of electron-dense cortical granules (arrows) was observed just beneath the oolemma of the MII oocytes with a DZP and MII oocytes in the control group (B, A). Microvilli are numerous and long on the oolemma in each group (A, B). MV: microvilli; CG: cortical granules; PVS: perivitelline space; O: oocyte. Scale bar (A, B): 500 nm.

**Figure 5:**
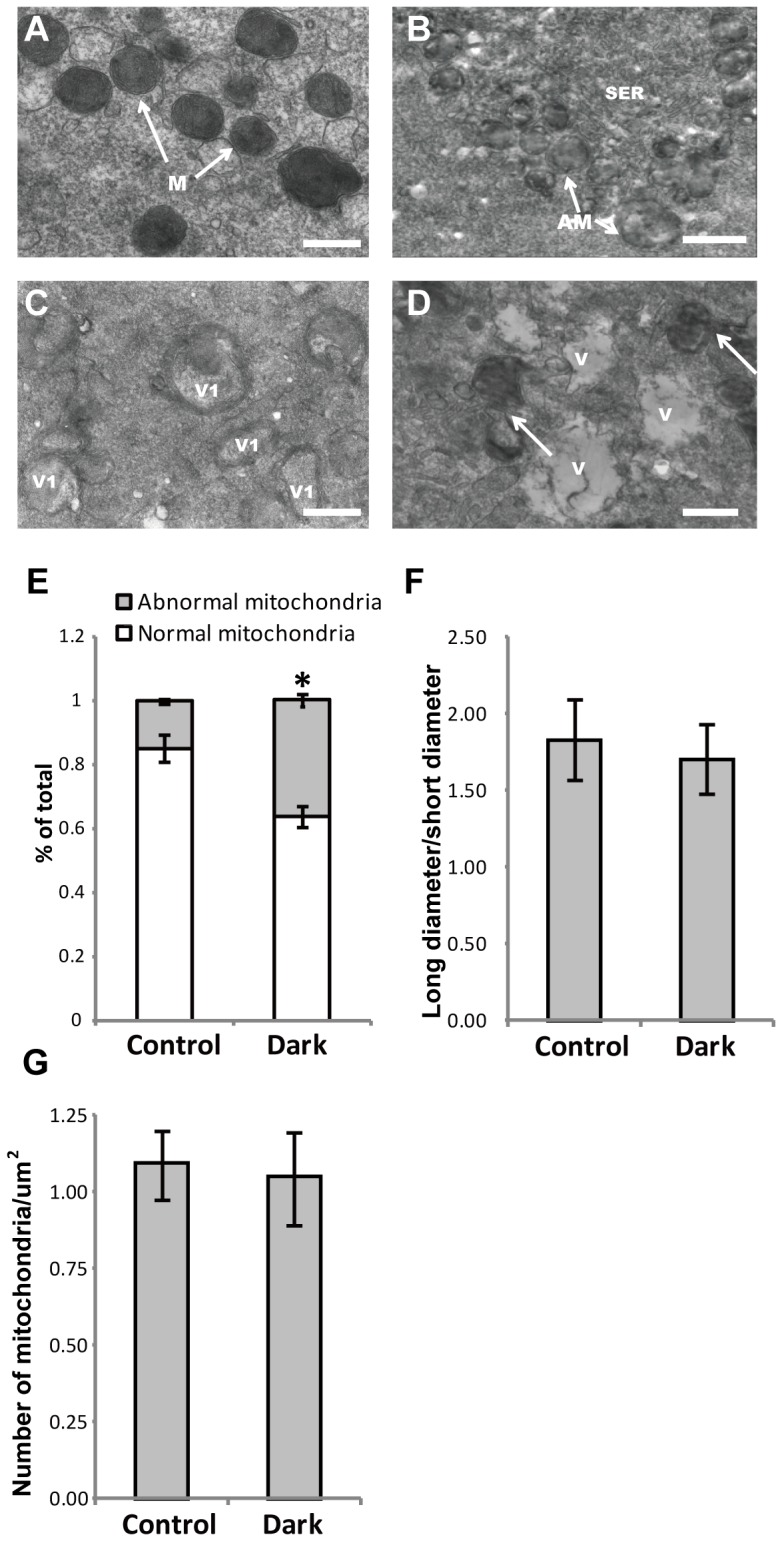
Ultrastructural differences in the mitochondria of normal versus oocytes with a dark zona pellucida, and the morphological characteristics and density of mitochondria in normal versus oocytes with a dark zona pellucida. The arched cristae were determined using electron microscopy (A). Mitochondrial cristae had a spherical or oval shape and presented arch-like cristae, which were located peripherally or transversely (arrows, A). In oocytes with a DZP, an intimate relationship between the SER and abnormal mitochondria was observed (arrows, B); numerous abnormal mitochondria with vesicle complexes (C); distribution and density of mitochondria were similar to the control. The whole part of the oolemma was filled with small and large vacuoles and abnormal mitochondria (arrows, D). M: Mitochondria; AM: abnormal mitochondria; V: vacuole; V1: vesicles encircled by flattened mitochondria; SER: smooth endoplasmic reticulum associated with mitochondria; Scale bar (A, B, C, and D): 500 nm. The rate of abnormal mitochondria was significantly lower in the control group (16.4 vs. 38.7%) (E). There were no significant differences between groups with long diameter/short diameter (1.73 vs. 1.62) and the density of mitochondria in each cytoplasm (1.16 vs. 1.12/µm2) (F and G). Bars indicated the standard deviation (SD) of the mean. *: compared with the control group, the abnormal mitochondria was significantly increased (P<0.05) in oocytes with a DZP. Note: Abnormal mitochondria rate: the number of abnormal mitochondria/total number of mitochondria; density of mitochondria: number of mitochondria/µm^2^.

A regular oolemma with numerous microvilli (Figures 4A and B) and rich peripherally aligned cortical granules in a continuous sub-oolemmal array were observed (Figures 4A and B). In oocytes with an NZP, small mitochondria-vesicle (MV) complexes were detected, and these normal mitochondria were predominantly spherical to oval in shape and presented an inter-looking appearance with a dense matrix and a few arch-like cristae, which were located peripherally or transversely (Fig. 5A). Typical mitochondria-smooth endoplasmic reticulum (M-SER) aggregates, in which anastomosing SER tubuli were surrounded by mitochondria that appeared abnormal with swollen and blurred shape in oocytes with a DZP were observed (Fig. 5B and C). However, the abnormal mitochondria observed in Fig. 5B and 5C appeared encircling or containing vacuoles, and large cytoplasmic vacuoles, a sign of early regression, were found in the DZP oocytes (Fig. 5D).

No significant differences were found between the oocytes with an NZP or DZP in the long or short diameter of the mitochondria (1.73 vs. 1.62) or in the density of total mitochondria (1.16/µm^2^ vs. 1.12/µm^2^) within the cytoplasm (Figures 5F and G). However, the percentage of abnormal mitochondria significantly increased in oocytes with a DZP compared to oocytes with an NZP (16.4% vs. 38.7%) (Figure 5E).

## Discussion

Irregularities in the composition, thickness or shape of the ZP have been shown to affect the fertilization, embryo quality and implantation rates [3], [18], [20], [21], [26]. In several previous studies on oocytes, which were surrounded by a DZP, no negative effects were found on the outcomes [23], [27]. However, in our study, the DZP was suggested to reflect the quality of the oocytes and the outcome of IVF/ICSI treatment. This discrepancy in interpretation could potentially be due to the transfer of a mix of embryos surrounded by both a DZP and NZP in the latter studies; however, in our study, we also incorporated couples in which we only transferred embryos that were surrounded by a DZP.

In group A, a mixture of embryos surrounded by both a dark and normal ZP was transferred, and there were no effects on the fertilization and implantation rate as well as the embryo quality, which were consistent with the outcome obtained in previous studies. In couples where all of the oocytes were surrounded by a DZP (Group B), there were no effects on fertilization in the IVF or the ICSI cycles, whereas the percentage of good quality embryos, implantation and clinical pregnancy rates were reduced.

Oocytes with a DZP contained abnormal mitochondria and a large number of vacuoles, whereas the distribution pattern of other organelles was not affected, such as the microvillus and cortical granules. Furthermore, normal mitochondria should be round or oval, containing few peripheral arch-like or transversal cristae and are usually closely surrounding the smooth endoplasmic reticulum (SER), whereas the cristae were replaced with mitochondria-vesicles and cyto-membrane depressions in the abnormal mitochondria from oocytes with a DZP. [28]–[30]. Mitochondria are the powerhouse of the oocyte [31], [32], and their activities are essential for normal oocyte maturation and developmental capacity [33], [34]. Moreover, studies on oocytes surrounded by DZP demonstrated that defects in the differentiation, morphology and inadequate redistribution of mitochondria might affect the metabolic activity of the oocyte, fertilization rate and developmental competency of the embryos [6], [29], [30], [35]–[37].

Scanning electron microscopy of the ultrastructure of the ZP in healthy mature human oocytes revealed a large number of fenestrations, which were formed by networked filaments [38]. In oocytes surrounded by a DZP, numerous high density materials were found, which most likely resulted in optical changes and thus presented a dark appearance in the ZP. Another prominent characteristic of these oocytes was the high incidence of vacuolization in their cytoplasm; thus, the DZP appeared to be correlated to defects within the oocytes. In fact, oocyte cytoplasmic vacuolization that are associated with the presence of damaged mitochondria, ruptures in the oolemma, decreased amount of microvilli, and characteristics of degenerating oocytes have been reported [39]. In addition, vacuoles are rare in mature and good quality oocytes, but may be found in cytoplasmically damaged, early regression, or aging oocytes [40]–[42]. In summary, the significantly decreased outcome, cytoplasmic vacuolization and damaged mitochondria in oocytes with a DZP suggested that the oocyte quality (such as cytoplasmic damage) was correlated with a DZP in IVF/ICSI cycles [6], [38], [40]–[42].

In this study, the percentage of 2PN was decreased in IVF cycles (59.4% vs. 76.3%) and ICSI cycles (67.7% vs. 82.3%) in Group B, which indicated that the increased closely packed electron-dense fibrillar materials in the DZP might not directly prevent sperm-egg binding. However, damaged mitochondria and significantly increased vacuoles have been reported as signs of cytoplasmic damage in mammalian oocytes [41], [42]. Moreover, oocytes with damaged cytoplasm are associated with decreased oocyte quality, low embryo development potential, and adverse pregnancy outcomes [41]–[45]. Thus, on the basis of the decreased rate of good quality embryos, implantation and clinical pregnancy rates in Group B and the ultrastructural analysis of oocytes with a DZP, we proposed that oocytes with a DZP might contain cytoplasmic damage, which may be the underlying reason for the decreased fertilization rate, rather than the denser texture in the DZP, which directly prevents sperm-egg binding [41], [42]. However, a role in the denser texture of the DZP in preventing sperm penetration at fertilization should also not be excluded.

In conclusion, oocytes surrounded by a DZP are associated with a decreased fertilization rate, low rate of good quality embryos, and adverse pregnancy outcome in IVF/ICSI cycles, as well as increased abnormal mitochondria and cytoplasmic vacuoles as detected using ultrastructural analysis. These outcomes suggest that the DZP could be used a selective marker for embryologists during daily laboratory work, and thus, we recommend avoiding the transfer of embryos derived from oocytes with a DZP.

## Supporting Information

Figure S1 **The thickness of the DZP was not increased compared with the normal ZP (16.27 vs. 16.97).**
(TIF)

## References

[1] Rienzi L, Ubaldi FM, Iacobelli M, Minasi MG, Romano S, et al (2008) Significance of metaphase II human oocyte morphology on ICSI outcome. Fertil Steril 90: 1692–1700. 18249393 10.1016/j.fertnstert.2007.09.024

[2] Montag M, Schimming T, Koster M, Zhou C, Dorn C, et al (2008) Oocyte zona birefringence intensity is associated with embryonic implantation potential in ICSI cycles. Reprod Biomed Online 16: 239–244. 18284880 10.1016/s1472-6483(10)60580-9

[3] Ebner T, Shebl O, Moser M, Sommergruber M, Tews G (2008) Developmental fate of ovoid oocytes. Hum Reprod 23: 62–66. 17977865 10.1093/humrep/dem280

[4] Ten J, Mendiola J, Vioque J, de Juan J, Bernabeu R (2007) Donor oocyte dysmorphisms and their influence on fertilization and embryo quality. Reprod Biomed Online 14: 40–48. 17207330 10.1016/s1472-6483(10)60762-6

[5] Otsuki J, Nagai Y, Chiba K (2007) Lipofuscin bodies in human oocytes as an indicator of oocyte quality. J Assist Reprod Genet 24: 263–270. 17653849 10.1007/s10815-007-9130-0 PMC3455008

[6] Familiari G, Heyn R, Relucenti M, Nottola SA, Sathananthan AH (2006) Ultrastructural dynamics of human reproduction, from ovulation to fertilization and early embryo development. Int Rev Cytol 249: 53–141. 16697282 10.1016/S0074-7696(06)49002-1

[7] Verlhac MH, Lefebvre C, Guillaud P, Rassinier P, Maro B (2000) Asymmetric division in mouse oocytes: with or without Mos. Curr Biol 10: 1303–1306. 11069114 10.1016/s0960-9822(00)00753-3

[8] Xia P (1997) Intracytoplasmic sperm injection: correlation of oocyte grade based on polar body, perivitelline space and cytoplasmic inclusions with fertilization rate and embryo quality. Hum Reprod 12: 1750–1755. 9308806 10.1093/humrep/12.8.1750

[9] Mikkelsen AL, Lindenberg S (2001) Morphology of in-vitro matured oocytes: impact on fertility potential and embryo quality. Hum Reprod 16: 1714–1718. 11473970 10.1093/humrep/16.8.1714

[10] Miao YL, Kikuchi K, Sun QY, Schatten H (2009) Oocyte aging: cellular and molecular changes, developmental potential and reversal possibility. Hum Reprod Update 15: 573–585. 19429634 10.1093/humupd/dmp014

[11] Alikani M, Palermo G, Adler A, Bertoli M, Blake M, et al (1995) Intracytoplasmic sperm injection in dysmorphic human oocytes. Zygote 3: 283–288. 8730892 10.1017/s0967199400002707

[12] Rienzi L, Balaban B, Ebner T, Mandelbaum J (2012) The oocyte. Hum Reprod 27 Suppl 1i2–21. 22811312 10.1093/humrep/des200

[13] Van Blerkom J, Henry G (1992) Oocyte dysmorphism and aneuploidy in meiotically mature human oocytes after ovarian stimulation. Hum Reprod 7: 379–390. 1587948 10.1093/oxfordjournals.humrep.a137655

[14] Shen Y, Stalf T, Mehnert C, Eichenlaub-Ritter U, Tinneberg HR (2005) High magnitude of light retardation by the zona pellucida is associated with conception cycles. Hum Reprod 20: 1596–1606. 15734754 10.1093/humrep/deh811

[15] Ebner T, Balaban B, Moser M, Shebl O, Urman B, et al (2010) Automatic user-independent zona pellucida imaging at the oocyte stage allows for the prediction of preimplantation development. Fertil Steril 94: 913–920. 19439291 10.1016/j.fertnstert.2009.03.106

[16] Liu C, Litscher ES, Mortillo S, Sakai Y, Kinloch RA, et al (1996) Targeted disruption of the mZP3 gene results in production of eggs lacking a zona pellucida and infertility in female mice. Proc Natl Acad Sci U S A 93: 5431–5436. 8643592 10.1073/pnas.93.11.5431 PMC39263

[17] Rankin TL, O’Brien M, Lee E, Wigglesworth K, Eppig J, et al (2001) Defective zonae pellucidae in Zp2-null mice disrupt folliculogenesis, fertility and development. Development 128: 1119–1126. 11245577 10.1242/dev.128.7.1119

[18] Loret De MolaJR, Garside WT, Bucci J, Tureck RW, Heyner S (1997) Analysis of the human zona pellucida during culture: correlation with diagnosis and the preovulatory hormonal environment. J Assist Reprod Genet 14: 332–336. 9226512 10.1007/BF02765837 PMC3454791

[19] Marco-Jimenez F, Naturil-Alfonso C, Jimenez-Trigos E, Lavara R, Vicente JS (2012) Influence of zona pellucida thickness on fertilization, embryo implantation and birth. Anim Reprod Sci 132: 96–100. 22607772 10.1016/j.anireprosci.2012.04.008

[20] Gabrielsen A, Lindenberg S, Petersen K (2001) The impact of the zona pellucida thickness variation of human embryos on pregnancy outcome in relation to suboptimal embryo development. A prospective randomized controlled study. Hum Reprod 16: 2166–2170. 11574510 10.1093/humrep/16.10.2166

[21] Palmstierna M, Murkes D, Csemiczky G, Andersson O, Wramsby H (1998) Zona pellucida thickness variation and occurrence of visible mononucleated blastomers in preembryos are associated with a high pregnancy rate in IVF treatment. J Assist Reprod Genet 15: 70–75. 9513844 10.1007/BF02766828 PMC3455425

[22] Balaban B, Urman B (2006) Effect of oocyte morphology on embryo development and implantation. Reprod Biomed Online 12: 608–615. 16790106 10.1016/s1472-6483(10)61187-x

[23] Esfandiari N, Burjaq H, Gotlieb L, Casper RF (2006) Brown oocytes: implications for assisted reproductive technology. Fertil Steril 86: 1522–1525. 16989830 10.1016/j.fertnstert.2006.03.056

[24] Dozortsev D, De Sutter P, Dhont M (1994) Behaviour of spermatozoa in human oocytes displaying no or one pronucleus after intracytoplasmic sperm injection. Hum Reprod 9: 2139–2144. 7868687 10.1093/oxfordjournals.humrep.a138406

[25] Nottola SA, Coticchio G, Sciajno R, Gambardella A, Maione M, et al (2009) Ultrastructural markers of quality in human mature oocytes vitrified using cryoleaf and cryoloop. Reprod Biomed Online 19 Suppl 317–27. 20034420 10.1016/s1472-6483(10)60280-5

[26] Liu DY, Baker HW (2000) Defective sperm-zona pellucida interaction: a major cause of failure of fertilization in clinical in-vitro fertilization. Hum Reprod 15: 702–708. 10686223 10.1093/humrep/15.3.702

[27] De Sutter P, Dozortsev D, Qian C, Dhont M (1996) Oocyte morphology does not correlate with fertilization rate and embryo quality after intracytoplasmic sperm injection. Hum Reprod 11: 595–597. 8671274 10.1093/humrep/11.3.595

[28] Nottola SA, Macchiarelli G, Coticchio G, Bianchi S, Cecconi S, et al (2007) Ultrastructure of human mature oocytes after slow cooling cryopreservation using different sucrose concentrations. Hum Reprod 22: 1123–1133. 17158818 10.1093/humrep/del463

[29] Au HK, Yeh TS, Kao SH, Tzeng CR, Hsieh RH (2005) Abnormal mitochondrial structure in human unfertilized oocytes and arrested embryos. Ann N Y Acad Sci 1042: 177–185. 15965061 10.1196/annals.1338.020

[30] Motta PM, Nottola SA, Makabe S, Heyn R (2000) Mitochondrial morphology in human fetal and adult female germ cells. Hum Reprod 15 Suppl 2129–147. 10.1093/humrep/15.suppl_2.129 11041520

[31] Balaban RS, Nemoto S, Finkel T (2005) Mitochondria, oxidants, and aging. Cell 120: 483–495. 15734681 10.1016/j.cell.2005.02.001

[32] Dumollard R, Duchen M, Carroll J (2007) The role of mitochondrial function in the oocyte and embryo. Curr Top Dev Biol 77: 21–49. 17222699 10.1016/S0070-2153(06)77002-8

[33] Nagano M, Katagiri S, Takahashi Y (2006) ATP content and maturational/developmental ability of bovine oocytes with various cytoplasmic morphologies. Zygote 14: 299–304. 17266788 10.1017/S0967199406003807

[34] Eichenlaub-Ritter U, Wieczorek M, Luke S, Seidel T (2011) Age related changes in mitochondrial function and new approaches to study redox regulation in mammalian oocytes in response to age or maturation conditions. Mitochondrion 11: 783–796. 20817047 10.1016/j.mito.2010.08.011

[35] Hoodbhoy T, Dean J (2004) Insights into the molecular basis of sperm-egg recognition in mammals. Reproduction 127: 417–422. 15047932 10.1530/rep.1.00181

[36] Keefe D, Tran P, Pellegrini C, Oldenbourg R (1997) Polarized light microscopy and digital image processing identify a multilaminar structure of the hamster zona pellucida. Hum Reprod 12: 1250–1252. 9222011 10.1093/humrep/12.6.1250

[37] Van Blerkom J (2004) Mitochondria in human oogenesis and preimplantation embryogenesis: engines of metabolism, ionic regulation and developmental competence. Reproduction 128: 269–280. 15333778 10.1530/rep.1.00240

[38] Nottola SA, Makabe S, Stallone T, Familiari G, Correr S, et al (2005) Surface morphology of the zona pellucida surrounding human blastocysts obtained after in vitro fertilization. Arch Histol Cytol 68: 133–141. 16079459 10.1679/aohc.68.133

[39] Motta PM, Nottola SA, Micara G, Familiari G (1988) Ultrastructure of human unfertilized oocytes and polyspermic embryos in an IVF-ET program. Ann N Y Acad Sci 541: 367–383. 3195921 10.1111/j.1749-6632.1988.tb22274.x

[40] Coticchio G, Borini A, Distratis V, Maione M, Scaravelli G, et al (2010) Qualitative and morphometric analysis of the ultrastructure of human oocytes cryopreserved by two alternative slow cooling protocols. J Assist Reprod Genet 27: 131–140. 20177770 10.1007/s10815-010-9394-7 PMC2854988

[41] El Shafie M, Sousa M, Windt M-L and Kruger TF. (2000) An Atlas of the Ultrastructure of Human Oocytes. Parthenon Publishing, New York, USA.

[42] Sá R, Cunha M, Silva J, Luís A, Oliveira C, et al (2011) Ultrastructure of tubular smooth endoplasmic reticulum aggregates in human metaphase II oocytes and clinical implications. Fertil Steril 96(1): 143–149. 21621206 10.1016/j.fertnstert.2011.04.088

[43] Silva RC, Báo SN, Jivago JL, Lucci CM (2011) Ultrastructural characterization of porcine oocytes and adjacent follicular cells during follicle development: lipid component evolution. Theriogenology 76(9): 1647–57. 21835450 10.1016/j.theriogenology.2011.06.029

[44] Windt ML, Coetzee K, Kruger TF, Marino H, Kitshoff MS, Sousa M, et al (2001) Ultrastructural evaluation of recurrent and in-vitro maturation resistant metaphase I arrested oocytes. Hum Reprod 16(11): 2394–8. 11679527 10.1093/humrep/16.11.2394

[45] Sousa M, da Silva JT, Silva J, Cunha M, Viana P, et al (2013) Embryological, clinical and ultrastructural study of human oocytes presenting indented zona pellucida. Zygote 2: 1–13. 10.1017/S0967199413000403 23992046

