# Supplementary figures and images for: Oocytes with a Dark Zona Pellucida Demonstrate Lower Fertilization, Implantation and Clinical Pregnancy Rates in IVF/ICSI Cycles
Source: PLoS One. 2014 Feb 24;9(2):e89409. doi: 10.1371/journal.pone.0089409 (PMC3933533; doi:10.1371/journal.pone.0089409)

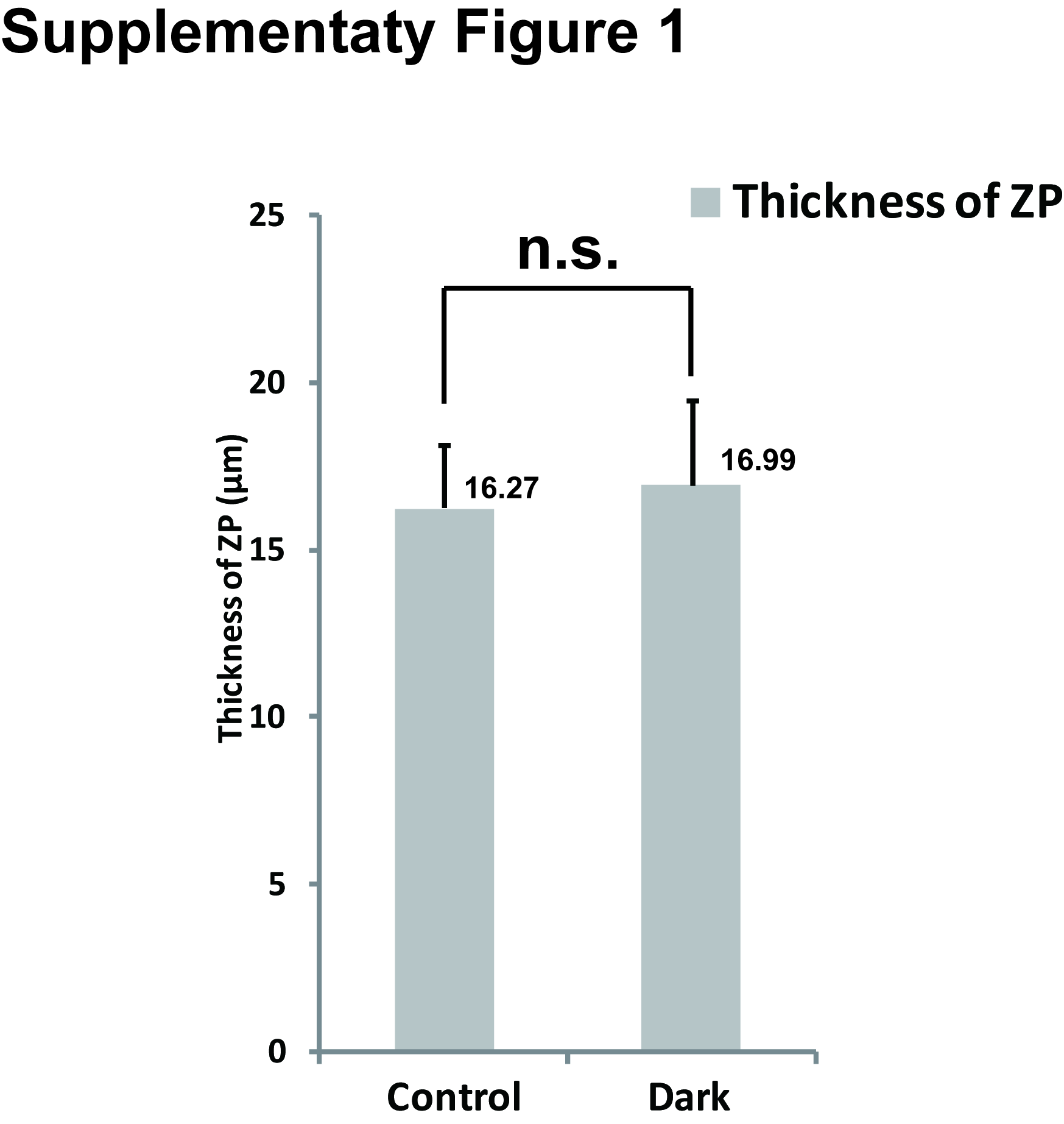

Supplement: Figure S1 — The thickness of the DZP was not increased compared with the normal ZP (16.27 vs. 16.97). (TIF) [file pone.0089409.s001.tif]
